# Reverse-D-4F improves endothelial progenitor cell function and attenuates LPS-induced acute lung injury

**DOI:** 10.1186/s12931-019-1099-6

**Published:** 2019-06-26

**Authors:** Nana Yang, Hua Tian, Enxin Zhan, Lei Zhai, Peng Jiao, Shutong Yao, Guohua Lu, Qingjie Mu, Juan Wang, Aihua Zhao, Yadong Zhou, Shucun Qin

**Affiliations:** 1 Experimental Center for Medical Research, Weifang Medical University, Weifang City, People’s Republic of China; 2 Key Laboratory of Atherosclerosis in Universities of Shandong, Institute of Atherosclerosis, Shandong First Medical University, Tai-an City, People’s Republic of China; 3 Institute of Preschool Education, Jinan Preschool Education College, Jinan City, People’s Republic of China; 4 Heart Center of Shandong First Medical University, Tai-an City, People’s Republic of China; 5 Department of Pharmaceutical Sciences, Binzhou Medical College, Yantai City, People’s Republic of China; 6 Department of Emergency Medicine, the second Affiliated Hospital of Shandong First Medical University, Tai-an City, People’s Republic of China

**Keywords:** Acute lung injury, Endothelial progenitor cells, Reverse D-4F, Apolipoprotein A-I, Endothelial nitric oxide synthase

## Abstract

**Background:**

Patients with acute lung injury (ALI) have increased levels of pro-inflammatory mediators, which impair endothelial progenitor cell (EPC) function. Increasing the number of EPC and alleviating EPC dysfunction induced by pro-inflammatory mediators play important roles in suppressing ALI development. Because the high density lipoprotein reverse-D-4F (Rev-D4F) improves EPC function, we hypothesized that it might repair lipopolysaccharide (LPS)-induced lung damage by improving EPC numbers and function in an LPS-induced ALI mouse model.

**Methods:**

LPS was used to induce ALI in mice, and then the mice received intraperitoneal injections of Rev-D4F. Immunohistochemical staining, flow cytometry, MTT, transwell, and western blotting were used to assess the effect of Rev-D4F on repairment of lung impairment, and improvement of EPC numbers and function, as well as the signaling pathways involved.

**Results:**

Rev-D4F inhibits LPS-induced pulmonary edema and decreases plasma levels of the pro-inflammatory mediators TNF-α and ET-1 in ALI mice. Rev-D4F inhibited infiltration of red and white blood cells into the interstitial space, reduced lung injury-induced inflammation, and restored injured pulmonary capillary endothelial cells. In addition, Rev-D4F increased numbers of circulating EPC, stimulated EPC differentiation, and improved EPC function impaired by LPS. Rev-D4F also acted via a PI3-kinase-dependent mechanism to restore levels of phospho-AKT, eNOS, and phospho-eNOS suppressed by LPS.

**Conclusions:**

These findings indicate that Rev-D4F has an important vasculoprotective role in ALI by improving the EPC numbers and functions, and Rev-D4F reverses LPS-induced EPC dysfuncion partially through PI3K/AKT/eNOS signaling pathway.

**Electronic supplementary material:**

The online version of this article (10.1186/s12931-019-1099-6) contains supplementary material, which is available to authorized users.

## Background

Acute lung injury (ALI) is a common clinical syndrome characterized by high mortality [1]. ALI pathogenesis can be caused by biological and abiotic pathogens. Biological pathogens include bacteria, viruses, fungi, atypical pathogens, and malignant tumors, while abiotic pathogens include acidic substances, drugs, toxic gas inhalation, and mechanical ventilation-related injuries [2]. Initially, endotoxins and other harmful substances in the blood circulation injure pulmonary capillary endothelial cells (PCEC), causing an increase in permeability in endothelial cell monolayers, as well as endothelial cell shrinkage and death. Approximately two hours post-injury, pulmonary interstitial edema can occur, and alveolar edema occurs approximately 12–24 h post-injury [3].

Endothelial cell dysfunction and inflammation play important roles in ALI initiation and development [3]. In ALI, an improved patient survival correlates with a higher colony count of endothelial progenitor cell (EPC) [4]. EPC engraftment maintains the integrity of pulmonary alveolar-capillary barrier, reestablishes endothelial function in vessels, and ameliorates the inflammatory state in LPS-induced ALI in rat models [5]. Therefore, EPC contributes to the repair of endothelial impairment and prevention of ALI development. However, patients with ALI have increased levels of pro-inflammatory mediators (e.g., ox-LDL, IL-6, TNF-α), which impair EPC function, including proliferation, migration, and tube formation. These impaired EPC are unable to restore the damaged endothelial system in patients with ALI [6]. Therefore, increasing the number of EPC and alleviating EPC dysfunction induced by pro-inflammatory mediators may suppress the development of ALI.

High-density lipoprotein (HDL) regulates EPC mobilization from bone marrow, prevents EPC apoptosis, and promotes differentiation of EPC into endothelial cells [7]. Apolipoprotein A-I (apoA-I), an important functional component of HDL, has 243 amino acids, and improves survival in septic rats [8]. Because the lipid-binding properties of apoA-I are largely related to its class A amphipathic helices, designed mimetic peptides, which contain four phenylalanine residues on the hydrophobic face (4F; F being the biochemical symbol for phenylalanine) and mimic the class A amphipathic helices, have no sequence homology with apoA-I [9]. The 4F residues have anti-inflammatory and antioxidant activities, and improve survival in septic rats [8]. Kruger et al. have shown that the mimetic peptide D-4F can prevent the loss of certain EPC functions by increasing endothelial nitric oxide synthase (eNOS) in EPC [10]. Our previous studies revealed that reverse D-4F (Rev-D4F) acts via a PI3K/AKT/eNOS-dependent mechanism to significantly improve EPC proliferation, migration, and tube formation [11]. Thus, HDL, apoA-I, and its mimetic peptides are new drugs for the treatment of lung injury [12]. However, the precise mechanisms of the protective effects of HDL and HDL mimetic peptides Rev-D4F on EPC damage induced by LPS have not been entirely clarified. In this study, we investigated the effect of Rev-D4F on EPC numbers and function in LPS-induced ALI mice.

## Methods

### Animals

Male C57 mice (6 weeks old) were obtained from the Vital River Company (Beijing, China) and randomly divided into four groups with six mice in each group: the control group (PBS), Rev-D4F group (Rev-D4F 10 mg/Kg and PBS), LPS group (PBS and LPS 10 mg/Kg), and Rev-D4F treatment group (Rev-D4F 10 mg/Kg and LPS 10 mg/Kg). Either vehicle or Rev-D4F (10 mg/kg) was administered by intraperitoneal injection.

All animal procedures complied with the Guide for the Care and Use of Laboratory Animals published by Weifang Medical University, and the protocol was approved by the Committee on the Ethics of Animal Experiments of Weifang Medical University. Mice were anesthetized by intraperitoneal injection of 400 mg/kg chloral hydrate.

### Rev-D4F synthesis

The Reverse D4F mimetic peptide synthesis method was used to obtain inverse chirality with D-amino acids only and reverse order of D4F (Ac-DWFKAFYDKVAEKFKEAF-NH2) via a practical approach of Fmoc solid-phase peptide synthesis by the Scilight-Peptide INC (Beijing, BJ, China). The structure and purity (98%) were determined by GC/MS.

### Analysis of wet: dry weight ratio

The left lung was taken, and lung surface liquid was absorbed with an absorbent paper. Weight of the left lung was considered the wet weight. The lung was dried at 60 °C for 48 h, and then reweighed to obtain the dry weight. The degree of lung edema was evaluated by calculating the wet: dry weight ratio.

### Measurement of serum cytokines

ELISA kits (Blue Gene, Shanghai, China) were used to measure TNF-α and ET-1 levels in mice plasma according to the manufacturer’s instructions.

### Histopathologic evaluations and Immunohistochemical staining

The right lobes of the lungs were fixed in 10% formalin (Sigma-Aldrich, St Louis, MO, USA), embedded in paraffin, cut to 6 μm sections, and then stained with Hematoxylin-eosin (Sigma-Aldrich, St Louis, MO, USA). Immunohistochemistry (IHC) was performed with specific antibodies against Sca-1 (Abcam, Cambridge, UK), VCAM-1, ICAM-1, eNOS, iNOS, or vWF (Santa Cruz Biotechnology, Santa Cruz, CA, USA), and the samples were then counterstained with Hematoxylin.

Red and white blood cells in the lung parenchyma were counted. Red and white blood cell counts, as well as quantitative alveolar wall thickness measurements were performed based on the segmented morphology in 10 randomly selected fields in each section (*n* = 6/group) using high power fields (× 200).

### Flow cytometric analysis of EPC

Red blood cell-lysing buffer (BD Biosciences, San Jose, CA, USA) was used to lyse peripheral blood erythrocytes. Then, other cells were labeled with the FITC-conjugated antibodies against CD34, PE-conjugated antibodies against FLK-1, and APC-conjugated antibodies against CD133 (BD Biosciences, San Jose, CA, USA). A gating strategy and isotype-identical antibodies were used to exclude debris and nonspecific fluorescent signals.

### EPC isolation

In vitro isolation of mice bone marrow-derived EPC were performed as described previously [11]. The mononuclear cells (MNCs) were obtained from the femurs of 4-week-old male C57 mice by density gradient centrifugation (Sigma-Aldrich, St Louis, MO, USA), and plated on fibronectin-coated 6 well plates in EGM-2MV (Endothelial cell basal medium-2, plus FBS, VEGF, R-IGF-1, rhEGF, rhFGF-B, GA-1000, hydrocortisone and ascorbic acid) (Lonza, Basel, Switzerland). After 3 days, non-adherent cells were removed, and fresh medium was applied every 3 days.

### Characterization of EPC

MNCs from mice bone marrow were cultured for 10 days, incubated with 2.5 mg/L DiI-ac-LDL (Thermo Fisher Scientific Inc., Waltham, MA, USA) for 2 h at 37 °C, and 10 mg/L FITC-UEA (Sigma-Aldrich, St Louis, MO, USA) for 1 h at 37 °C, and then fixed for 5 min with 1–2% paraformaldehyde (Sigma-Aldrich, St Louis, MO, USA). Double-positive cells and total cell numbers were counted in five random fields under a fluorescence microscope (× 100). The positive cells (%) represent the numbers of double-positive cells compared with the total number of cells.

### EPC treatment

MNCs obtained from mice bone marrow were induced for 21 days by EGM-2MV and differentiated into late outgrowth EPC. Before experiment, the cell medium was changed to basic medium (M199 + 5% FBS), and cells were separated into 5 groups and treated as follows: Control (M199 + 5% FBS), Rev-D4F (M199 + 5% FBS + 50 μg/ml reverse D-4F), LPS (M199 + 5% FBS + 30 μg/ml LPS), LPS + Rev-D4F (M199 + 5% FBS + 50 μg/ml reverse D-4F + 30 μg/ml LPS), LPS + Rev-D4F + LY294002 (M199 + 5% FBS + 50 μg/ml reverse D-4F + 30 μg/ml LPS + 30 μM LY294002). After pretreatment with the PI3-kinase inhibitor LY294002 (30 μM) (Sigma-Aldrich, St Louis, MO, USA) for 2 h, EPC were incubated with Rev-D4F (50 μg/ml) for 6 h, and then treated with LPS for 36 h to induce EPC damage without removal of LY294002 and Rev-D4F.

### EPC viability and proliferation

EPC viability was determined by MTT assay. After different treatments, cells were incubated with 20 μl 5 mg/ml MTT (Sigma-Aldrich, St Louis, MO, USA) for 4 h at 37 °C. The supernatant was aspirated, and 150 μL of dimethyl sulfoxide (DMSO, Sigma-Aldrich, St Louis, MO, USA) was added to each well and shaken for 10 min. OD values were measured at 490 nm.

EPC proliferation was evaluated by flow cytometry analysis. After treatments, cells were digested with 0.25% trypsin (Sigma-Aldrich, St Louis, MO, USA), fixed with 75% ethanol, and stained with FITC-conjugated antibodies against Ki67 (BD Biosciences, San Jose, CA, USA). A gating strategy and isotype-identical antibodies were used to exclude debris and nonspecific fluorescent signals.

### EPC migration

EPC migration was measured using an 8 μm pore 24-well Cell Migration Assay kit (BD Biosciences, San Jose, CA, USA). After treatments, cells were digested with 0.25% trypsin, and 1.2 × 10^4^ cells in M199 were placed in the upper chamber. EGM-2MV medium was placed in the lower compartment. After 24 h incubation at 37 °C, the upper cells were removed with a cotton wool swab, and the transwell filters were fixed and stained with DAPI (Sigma-Aldrich, St Louis, MO, USA). The migratory cells were counted in five random microscopic fields (× 100).

### EPC adhesion

Mice bone marrow-derived MNCs were cultured on fibronectin-coated 6 well plates at 10^6^ cells per cm^2^. After 3 days, non-adherent cells were removed, and adherent cells were cultured for additional 4 days. Then, we randomly selected five fields of view and counted the number of adherent cells under a microscope (× 100).

### Tube formation in vitro

After different treatments, cells were digested with 0.25% trypsin and 1.0 × 10^4^ cells in M199 plus 10% FBS were placed on matrigel (BD Biosciences, San Jose, CA, USA) in a 96 wells plate. After 18 h incubation, the average total length of complete tubes formed by cells was measured under a microscope (× 40) in five random microscopic fields using computer software, Image-Pro Plus.

### Western blot analysis

Total protein was extracted with RIPA lysis buffer and quantified using the BCA method. In total, 30 μg of protein was electrophoresed on a 10% denaturing polyacrylamide gel, and transferred onto PVDF membranes. After blocking with 5% dried skimmed milk, the membranes were incubated with antibodies against β-actin (1: 20000, Sigma-Aldrich, St Louis, MO, USA), AKT (1: 2000, Cell Signaling Technologies, Beverly, MA, USA), phosphor-AKT (1: 1000, Cell Signaling Technologies, Beverly, MA, USA), eNOS (1: 500, Santa Cruz Biotechnology, Santa Cruz, CA, USA), phosphor-eNOS (1: 1000, Cell Signaling Technologies, Beverly, MA, USA), CD133 (1: 500, Abcam, Cambridge, UK), FLK-1 (1: 500,Abcam, Cambridge, UK) or Sca-1 (1: 500, Abcam, Cambridge, UK) for 3 h, and subsequently incubated with horseradish peroxidase-conjugated goat anti-rabbit IgG antibody (1: 3000, Santa Cruz Biotechnology, Santa Cruz, CA, USA). The signals were detected using the Phototope-HRP Western Detection Kit (Thermo Fisher Scientific Inc., Waltham, MA, USA).

### Immunofluorescence staining

MNCs were cultured and differentiated into relatively mature endothelial cells. The lung tissues were used to make frozen sections. Next, cells and frozen sections were blocked with goat serum at room temperature for 30 min, and then incubated with anti-mouse CD31 (Santa Cruz Biotechnology, Santa Cruz, CA, USA), vWF (Santa Cruz Biotechnology, Santa Cruz, CA, USA), CD133 (Abcam, Cambridge, UK) or FLK-1 (Abcam, Cambridge, UK) antibodies for 1 h at 37 °C. Finally, cells and tissues were incubated with secondary antibodies conjugated with Cy3 or FITC, and staining was assessed under fluorescence microscopy (× 200).

### Statistical analysis

All data are presented as mean ± SD. Variables between groups were assessed by one-way ANOVA using the software program SPSS11.5. Values of *P*<0.05 were considered significant.

## Results

### Rev-D4F suppresses LPS-induced lung injury in ALI mice

**Fig. 1:**
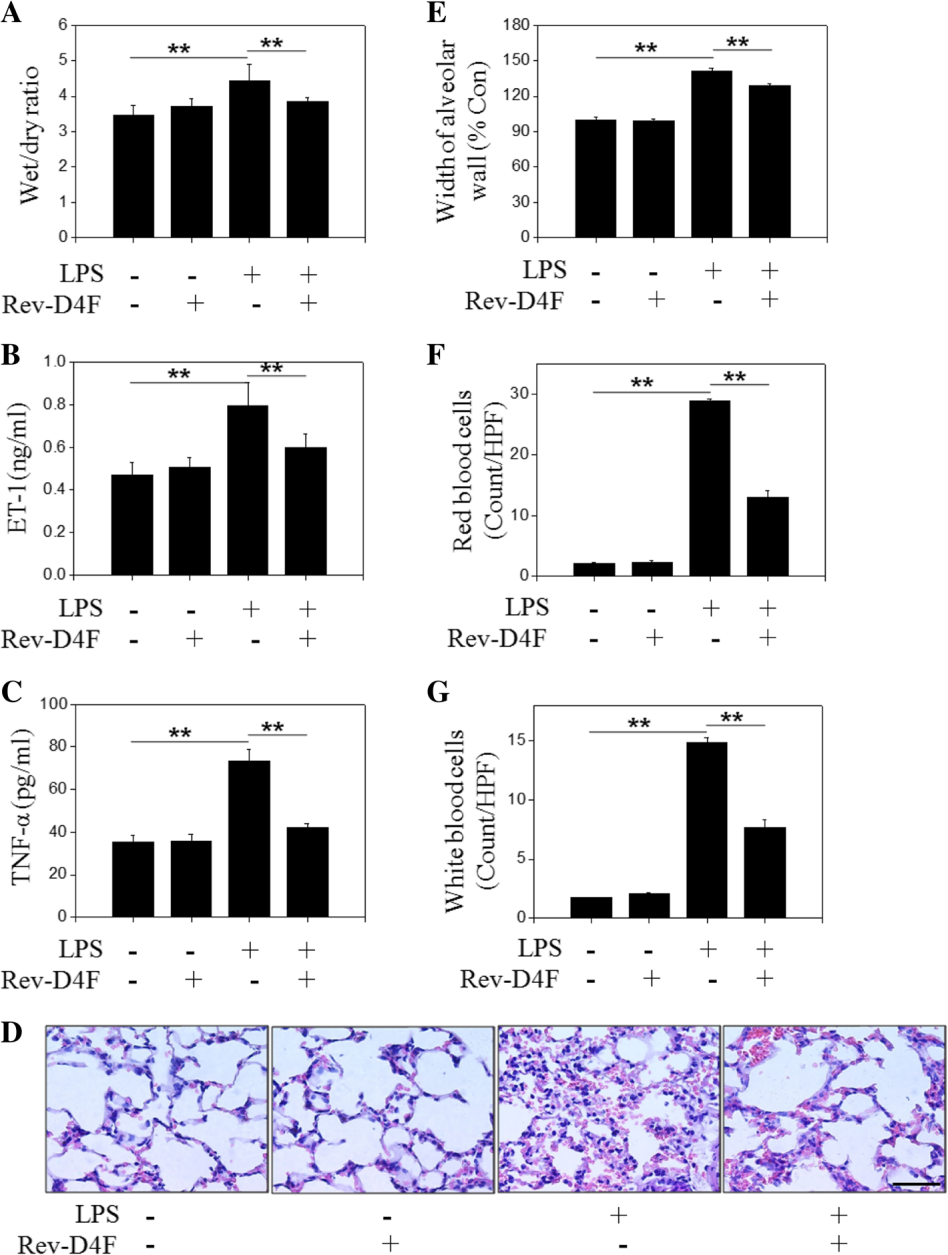
Rev-D4F suppresses LPS-induced lung injury in endotoxemic mice. Mice were treated with LPS and/or Rev-D4F for 48 h, sacrificed, and the left lung lobes were used to determine the wet: dry ratio **a** The plasma was used to detect the levels of ET-1 **b** and TNF-α **c** The right lobes of the lungs were stained with Hematoxylin-eosin (× 200); the scale bar represents 50 μm **d** The alveolar wall thickness was quantified **e** The numbers of red **f** and white blood cells **g** were analyzed. Data are presented as mean ± SD (*n* = 6; **P < 0.01)

Compared to control group, LPS induced pulmonary edema, which was significantly inhibited by Rev-D4F in ALI mice (Fig. 1a). The pro-inflammatory cytokine TNF-α and the vasoconstrictor endothelin-1 (ET-1) contribute to ALI pathogenesis. Accordingly, LPS increased ET-1 and TNF-α levels in ALI mice plasma, which were inhibited by Rev-D4F (Fig. 1b, c).

In order to assess pulmonary injury, hematoxylin-eosin was used to stain lung tissues. The alveolar walls were thickened, and the interstitial space was infiltrated with red and white blood cells in the LPS-induced group (Fig. 1d-g). The majority of white blood cells were observed in the septa, and fewer red and white blood cells were observed in the alveolar spaces. Importantly, Rev-D4F reversed the LPS-induced changes (Fig. 1d-g).

### Rev-D4F reduces inflammation and restores injured lung PCEC in ALI mice

**Fig. 2:**
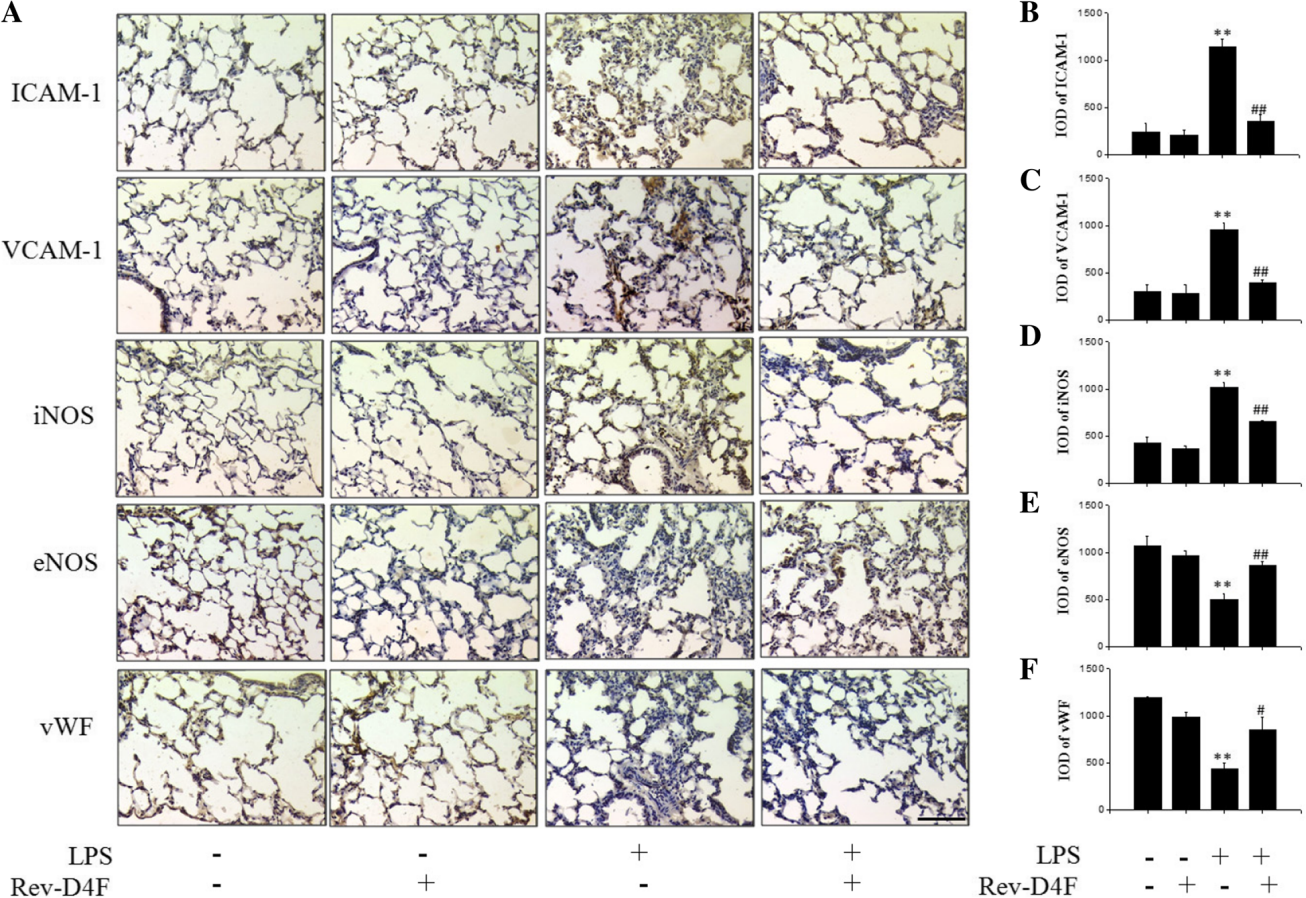
Rev-D4F reduces lung injury-induced inflammation and restores injured PCECs. **a** IHC of inflammatory markers (ICAM-1, VCAM-1, and iNOS) and endothelial cell surface markers (eNOS and vWF) under a microscope (× 200). Scale bar represents 50 μm. **b-f** Densitometric evaluation of the IHC images shown in panel A. Data are presented as mean ± SD (n = 6; **P < 0.01 versus NS, ^*#*^*P* < 0.05, ^*##*^*P* < 0.01 versus LPS group)

ICAM-1, VCAM-1, and iNOS are considered inflammation markers of endothelial injury [13], while eNOS and vWF are important surface markers of endothelial cells [14]. Compared with the control group, expression of ICAM-1, VCAM-1, and iNOS in lung tissue was significantly increased in the LPS treatment group. Notably, the levels of ICAM-1, VCAM-1, and iNOS were inhibited by Rev-D4F (Fig. 2).

The function of PCECs is often impaired in endotoxin-induced ALI, resulting in increased permeability of PCECs, and pulmonary interstitial and alveolar edema [3]. Importantly, Rev-D4F restored the levels of endothelial cell surface markers, eNOS and vWF, compared with the LPS-treated group (Fig. 2).

### Effect of rev-D4F on EPC numbers

**Fig. 3:**
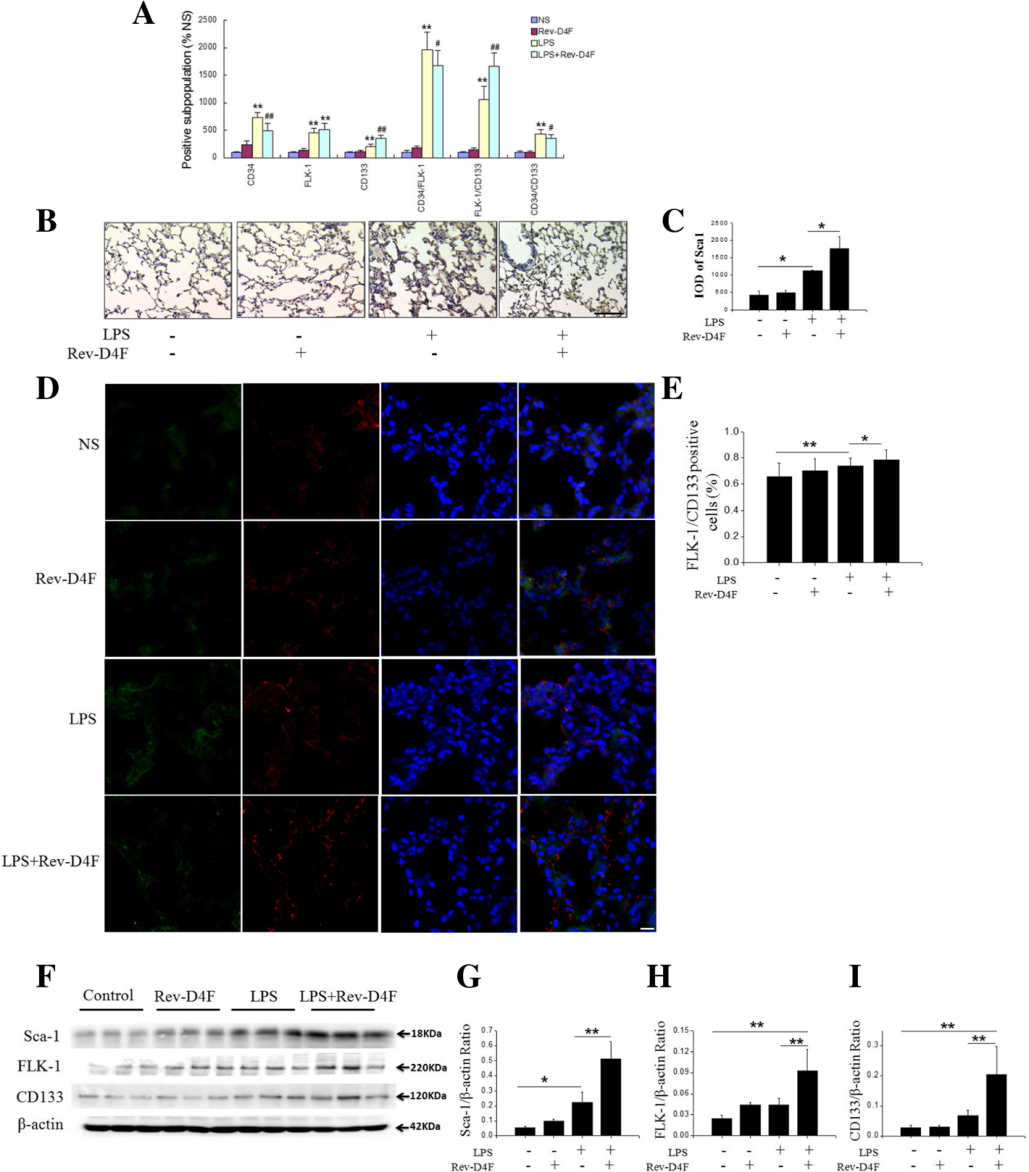
Effect of Rev-D4F on EPC numbers. **a** The number of CD34^+^ cells, FLK-1^+^ cells, CD133^+^ cells, CD34^+^/FLK-1^+^ cells, FLK-1^+^ /CD133^+^ cells, and CD34^+^/CD133^+^ cells from mice peripheral blood in NS group as a percentage of the control. Data are presented as mean ± SD (n = 6; **P < 0.01 versus NS, ^*#*^*P* < 0.05, ^*##*^*P* < 0.01 versus LPS group). **b** IHC of Sca-1^+^ cells in the right lobes of the lungs; scale bar represents 50 μm. **c** Densitometric evaluation of the data shown in panel B. **d** The FLK-1^+^/CD133^+^ double positive cells were detected by immunofluorescence staining (× 400); scale bar represents 20 μm. **e** The percentage of CD34^+^/FLK-1^+^ positive cells shown in panel D. **f** Western blot analysis of Sca-1, FLK-1, and CD133 protein levels in lung tissues. **g-i** Densitometric evaluation of the western blotting data shown in panel **f** (*P < 0.05, **P < 0.01)

EPC numbers positively correlate with the prognosis of patients with ALI [4]. Therefore, EPC mobilization contributes to the treatment of ALI. Compared with the control group, LPS significantly increased the numbers of CD34^+^, FLK-1^+^, CD133^+^, CD34^+^/FLK-1^+^, FLK-1^+^/CD133^+^, and CD34+/CD133^+^ cells in mice peripheral blood (Fig. 3a). Compared with the LPS treatment group, Rev-D4F inhibited the levels of CD34^+^, CD34/FLK-1^+^, and CD34^+^/CD133^+^ cells, while it increased the levels of FLK-1^+^, CD133^+^, and FLK-1^+^/CD133^+^ cells (Fig. 3a). The stem cells antigen-1 (Sca-1) has been used as a marker of EPC. Compared with control group, the number of Sca-1^+^ and FLK-1^+^/CD133^+^ cells was significantly increased in LPS-treated mice. Interestingly, Rev-D4F further increased the numbers of Sca-1^+^ and FLK-1^+^/CD133^+^ cells in lung tissues compared to LPS group (Fig. 3b-e). Compared with control group, the level of Sca-1 in lung tissues of LPS-treated mice increased significantly, while the level of FLK-1 and CD133 increased slightly (Fig. 3f-i).

### Rev-D4F restores EPC function in ALI mice

**Fig. 4:**
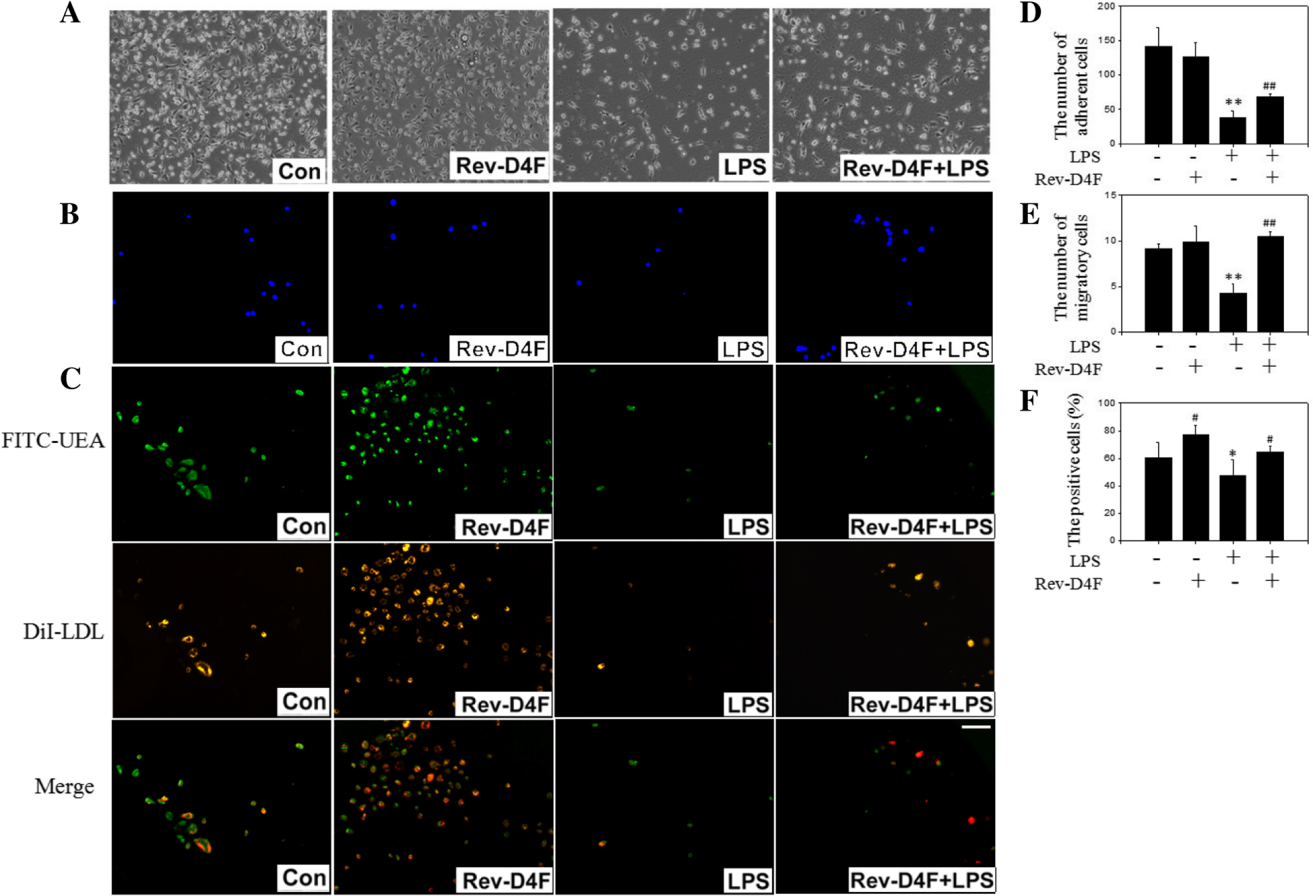
Effect of Rev-D4F on EPC function. MNCs derived from mice bone marrow were cultured to analyze the EPC function of adhesion (**a** × 100 and **d**), migration (**b** × 100 and **e**) and differentiation (**c** × 100 and **f**). Scale bar represents 100 μm. Data are presented as mean ± SD (*n* = 6); *P < 0.05, **P < 0.01 versus control group, ^*#*^*P < 0.05*, ^*##*^*P < 0.01* versus LPS group.

Compared with the control group, more EPC were observed in the LPS treatment group. In contrast, EPC functions, such as differentiation, migration, and adherence were impaired by LPS (Fig. 4). However, the impaired adherence and migration of EPC in the LPS treatment group were reversed by Rev-D4F (Fig. 4). Because EPC take up LDL and bind lectin (Fig. 4), these activities were considered as differentiation markers of EPC. The double-positive cell numbers decreased in LPS treatment group compared with control group, which was improved by Rev-D4F (Fig. 4).

### Characterization of EPC

After 14 days of culture, MNCs isolated from mouse bone marrow showed cobblestone-like morphology (Additional file 1: Figure S1). These induced MNCs took up LDL and bound lectin (Additional file 1: Figure S1). When cultured continuously, MNCs showed the characteristics of mature ECs, expressing CD31 and vWF as identified by immunofluorescence (Additional file 1: Figure S1). Therefore, these induced MNCs were characterized as differentiated EPC.

### LPS impairs EPC function

**Fig. 5:**
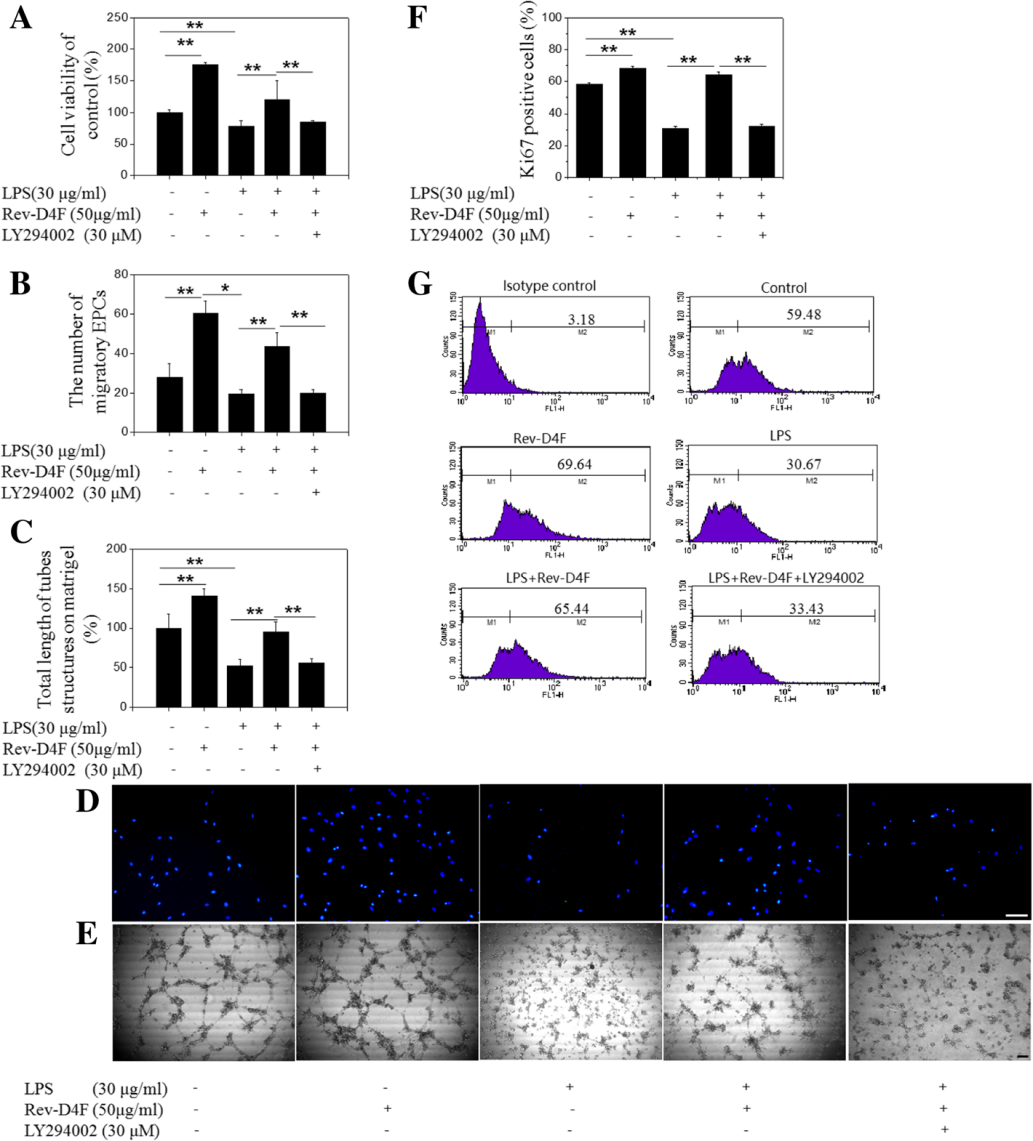
Rev-D4F alleviates EPC dysfunction induced by LPS. EPC were pretreated with the PI3-kinase inhibitor LY294002 (30 μM) for 2 h, and then incubated with reverse D-4F (50 μg/ml) for 6 h, followed by treatment with LPS for 36 h to induce EPC damage. Viability of EPC were assessed by MTT assay (**a**). EPC migration was measured by transwell assay, and the migratory cells were counted in five random microscopic fields (× 10) under fluorescence microscopy (**b** and **d** × 100). Treated EPC were plated on matrigel in a 96-well plate for 18 h, and the total length of tubules (% of control) was compared in each group (**c** and e × 40). Proliferation of EPC were assessed by Ki67 staining (**f** and **g**). Scale bar represents 100 μm. Data are means ± SD from at least three independent experiments; *P < 0.05, **P < 0.01

Pro-inflammatory mediators, such as LPS or TNF-α, damage the function of vascular wall cells [15]. Thus, we investigated the effect of LPS (0–30 μg/ml; 36 h) on EPC proliferation. We found that LPS significantly reduced proliferation (Additional file 2: Figure S2) and inhibited the EPC cycle in G1/S phase (Additional file 2: Figure S2). We also found that LPS significantly reduced EPC migration and tube formation (Fig. 5).

### Rev-D4F reverses LPS-induced impairment of EPC function

Previously, we found that Rev-D4F (25–100 μg/ml) could improve proliferation, migration, and tube formation of EPC [11]. To investigate whether Rev-D4F could reverse the LPS-induced impairment of EPC function, cells were pretreated with Rev-D4F for 6 h, incubated with LPS for 36 h, and their proliferation, migration, and tube formation in vitro were assessed. We found that Rev-D4F rescued the impairment of EPC function induced by LPS (Fig. 5).

### LY294002 inhibits rev-D4F-mediated restoration of EPC function

To investigate the protective mechanism of Rev-D4F, EPC were pre-incubated (2 h) with LY294002, a PI3-kinase inhibitor, followed by 6 h incubation with Rev-D4F, and stimulation with LPS. As shown in Fig. 5, inhibition of PI3-kinase activity by LY294002 inhibited the Rev-D4F-mediated restoration of LPS-induced EPC dysfunction (Fig. 5).

### Rev-D4F restores LPS-Iimpaired PI3K/AKT/eNOS pathway

**Fig. 6:**
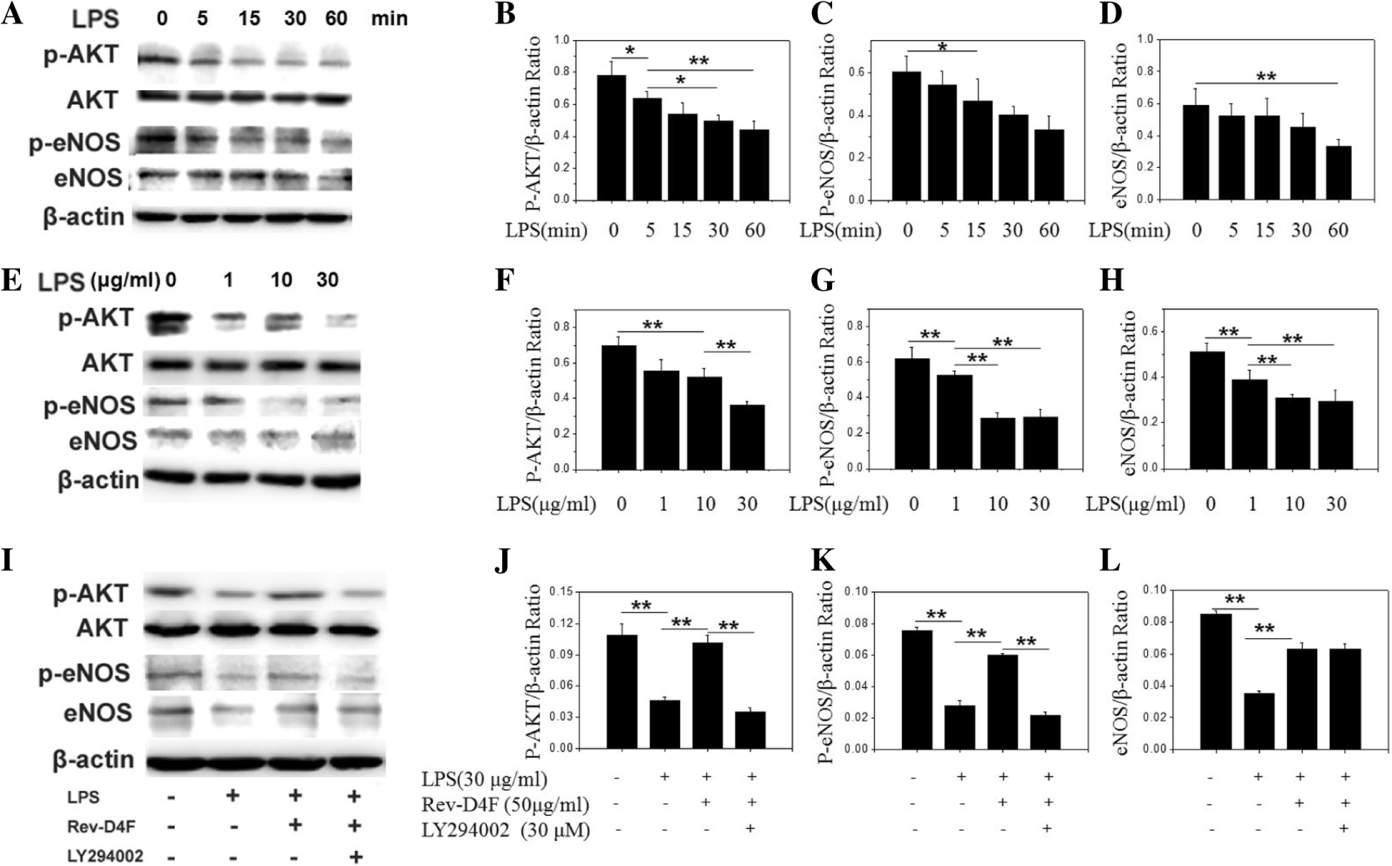
Effect of Rev-D4F and LPS on phosphor-AKT, eNOS, and phosphor-eNOS levels. Western blot analysis and densitometric evaluation of phosphor-AKT, eNOS, and phosphor-eNOS at different time points during treatment with LPS (30 μg/ml) (**a**-**d**) and with different LPS concentrations (0–30 μg/ml) (**e**-**h**). Western blot analysis and densitometric evaluation of phosphor-AKT, eNOS, and phosphor-eNOS in EPC treated with LPS, LPS with Rev-D4F, or LPS with Rev-D4F and LY294002 (**i**-**l**). *P < 0.05, **P < 0.01

The PI3K/AKT/eNOS pathway plays an important role in the biological functions of EPC, such as proliferation, migration, differentiation, and tube formation. Here we investigated the influence of Rev-D4F on the PI3K/AKT/eNOS signaling pathway. As shown in Fig. 6, LPS decreased the levels of phosphor-AKT, eNOS, and phosphor-eNOS. Pretreatment with Rev-D4F restored the levels of phosphor-AKT, eNOS, and phosphor-eNOS. Compared to cells treated with Rev-D4F, LY294002 decreased the phosphor-AKT and phosphor-eNOS protein levels (Fig. 6), indicating that the restorative effect of Rev-D4F on AKT and eNOS signaling in EPC is mediated by the PI3K pathway.

## Discussion

EPC were first isolated from peripheral blood using magnetic microbeads [15]. EPC contributes to physiological neovascularization, wound healing in vessels, tissue regeneration in ischemia [16], and repair of lung injury [17]. Our present findings demonstrate that Rev-D4F inhibits LPS-induced pulmonary edema, decreases plasma levels of TNF-αand ET-1, inhibits infiltration of red and white blood cells into the interstitial space, reduces injury-induced lung inflammation, and restores injured PCEC. Our previous study indicated that Rev-D4F improved EPC proliferation, migration, tube formation, and NO-releasing function, which was partially dependent on the PI3K/AKT/eNOS/NO pathway [11]. Our present results demonstrate that Rev-D4F improves the function of EPC impaired by LPS both in vivo and in vitro, and that Rev-D4F increases EPC numbers to repair injured PCEC.

The protective effects of apoA-I mimetic peptide 4F on vascular function in endotoxemic rats have been demonstrated. The anti-inflammatory effects of apolipoprotein A-I mimetic peptide (L-4F) in ARDS are due to the direct inhibition of endotoxin activity and increase of HDL antioxidant activity [18–20]. Similarly, our results demonstrate that another apoA-I mimetic peptide, Rev-D4F, repairs LPS-induced lung damage in mice. In addition, our results show that Rev-D4F increases numbers of EPC, both in peripheral blood and in lung tissues, and improves EPC function in LPS-treated mice.

Chronic inflammation decreases numbers of circulating EPC and suppresses their activity, resulting in suppressed restoration of vessel injury and continuous inflammation [21]. Reduced numbers of circulating EPC are associated with hypercholesterolemia [22], inflammation [23], obesity [24], and diabetes [25]. HDL, active in anti-inflammation and anti-oxidation, increases numbers of circulating EPC and promotes re-endothelialization in wound healing via the PI3K/Akt/cyclin D1 signaling pathway. In addition, HDL slows down EPC senescence by increasing NO and promoting telomerase activity via the PI3K/Akt signaling pathway [26]. ApoA-I, the major structural apolipoprotein of HDL, increases numbers of circulating EPC, promotes endothelial regeneration, and attenuates neointima formation in a murine model of transplant arteriosclerosis [27]. ApoA-I mimetic peptide D-4F, mimicking ApoA-I class A amphipathic helices, promotes EPC proliferation, migration, and adhesion [28]. Our previous study has indicated that reverse D-4F, a novel ApoA-I mimetic peptide, improves EPC proliferation, migration, tube formation, and NO-releasing function, partially through the PI3K/AKT/eNOS/NO pathway [11]. Few studies have investigated whether an ApoA-I mimetic peptide D-4F, especially reverse D-4F, exerts protective effects against EPC injury induced by LPS, and little is known about the potential mechanism.

In this study, we have found that LPS significantly impairs EPC functions, such as proliferation, migration, and tube formation, and Rev-D4F inhibits the LPS-induced EPC dysfunction. Patruno et al. have found that LPS reduces the phosphorylation levels of AKT in H9c2 cells, and that AKT is an important determinant of eNOS phosphorylation at Ser1177 [29]. PI3K/Akt activation plays an essential role in HDL-induced EPC proliferation, migration, and angiogenesis [30]. Our western blot results show that LPS reduces levels of eNOS, Ser1177-phosphor-eNOS, and phosphor-AKT in EPC, and that Rev-D4F restores the LPS-attenuated levels of these proteins. In addition, our results demonstrate that the PI3K/AKT inhibitor LY294002 inhibits the Rev-D4F-restorative effects on phosphor-AKT and phosphor-eNOS levels, indicating that the Rev-D4F-mediated restoration of EPC function in LPS-treated cells is mediated by PI3K/AKT /eNOS signaling pathway.

## Conclusions

Our results demonstrate that Rev-D4F repairs LPS-induced lung damage and reverses the LPS-induced EPC dysfunction. Although the mechanism of improving EPC function by Rev-D4F is not well understood, our results support the hypothesis that the restorative function of Rev-D4F is mediated by PI3K/AKT/eNOS signaling. Together, our data suggest that by improving the EPC function and numbers, Rev-D4F has an important vasculoprotective role in lung inflammatory injury.

## Additional files

Additional file 1: **Figure S1.** Immunofluorescence identification of bone marrow derived- EPC. MNCs from mice bone marrow cultured for 14 days showed endothelial cell-like morphology (A× 100), took up DiI-ac-LDL (B× 100), and bound lectin (C× 100). EPC, cultured for 10 days and differentiated, expressed mature endothelial markers, such as CD31 (D× 200) and vWF (E× 200), which were identified by immunofluorescence under fluorescence microscopy. Scale bar (B and C) represented 100 μm, and Scale bar (A, D and E) represented 50 μm. (PDF 114 kb)

Additional file 2: **Figure S2.** (A): The effect of different concentrations of LPS on EPC viability. **P < 0.01 versus LPS (0 μg/ml). (B): The effect of LPS on EPC was determined by cell cycle assessment. (PDF 107 kb)

## Data Availability

All data are provided in the manuscript.

## Abbreviations

ALI: Acute lung injury
ARDS: Acute respiratory distress syndrome
EPC: Endothelial progenitor cell
HDL: High density lipoprotein
LPS: Lipopolysaccharide
PCEC: Pulmonary capillary endothelial cells
