# Supplementary material for: Reverse-D-4F improves endothelial progenitor cell function and attenuates LPS-induced acute lung injury
Source: Respir Res. 2019 Jun 26;20:131. doi: 10.1186/s12931-019-1099-6 (PMC6595601; doi:10.1186/s12931-019-1099-6)

Additional file 1: Immunofluorescence identification of bone marrow derived- EPC. MNCs from mice bone marrow cultured for 14 days showed endothelial cell-like morphology (A×100), took up DiI-ac-LDL (B×100), and bound lectin (C×100). EPC, cultured for 10 days and differentiated, expressed mature endothelial markers, such as CD31 (D×200) and vWF (E×200), which were identified by immunofluorescence under fluorescence microscopy. Scale bar (B and C) represented 100μm, and Scale bar (A, D and E) represented 50μm.

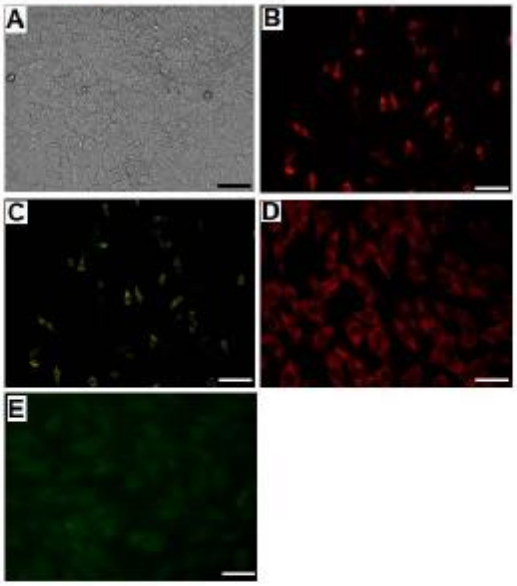

Supplement: Supplementary file 1 — Figure S1. Immunofluorescence identification of bone marrow derived- EPC. MNCs from mice bone marrow cultured for 14 days showed endothelial cell-like morphology (A× 100), took up DiI-ac-LDL (B× 100), and bound lectin (C× 100). EPC, cultured for 10 days and differentiated, expressed mature endothelial markers, such as CD31 (D× 200) and vWF (E× 200), which were identified by immunofluorescence under fluorescence microscopy. Scale bar (B and C) represented 100 μm, and Scale bar (A, D and E) represented 50 μm. (PDF 114 kb) [file 12931_2019_1099_MOESM1_ESM.pdf]
