# Supplementary material for: Reverse-D-4F improves endothelial progenitor cell function and attenuates LPS-induced acute lung injury
Source: Respir Res. 2019 Jun 26;20:131. doi: 10.1186/s12931-019-1099-6 (PMC6595601; doi:10.1186/s12931-019-1099-6)

Additional file 2: (A): The effect of different concentrations of LPS on EPC viability. **P <0.01 versus LPS (0μg /ml). (B): The effect of LPS on EPC was determined by cell cycle assessment.

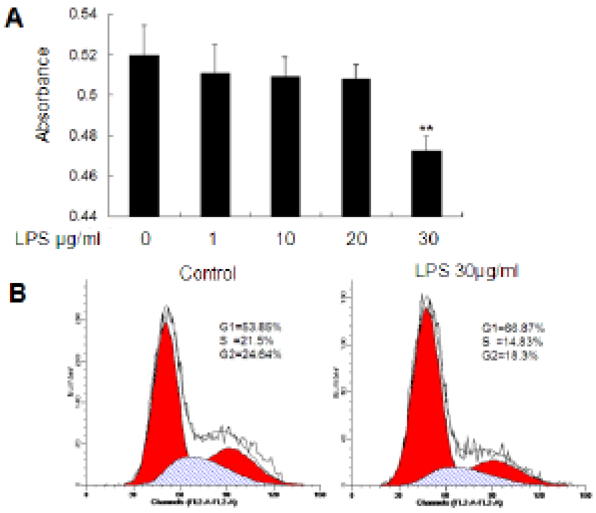

Supplement: Supplementary file 2 — Figure S2. (A): The effect of different concentrations of LPS on EPC viability. **P < 0.01 versus LPS (0 μg/ml). (B): The effect of LPS on EPC was determined by cell cycle assessment. (PDF 107 kb) [file 12931_2019_1099_MOESM2_ESM.pdf]
